# Editorial: Phenotypic variation as an important aspect of staphylococcal pathogenesis

**DOI:** 10.3389/fcimb.2024.1456380

**Published:** 2024-07-19

**Authors:** Agnieszka Bogut, Agnieszka Magryś

**Affiliations:** Chair and Department of Medical Microbiology, Medical University of Lublin, Lublin, Poland

**Keywords:** siderophores, cardiac implantable electronic device, staphylococcus, small colony variants, SCV (small colony variant), Manuka honey, phenotypic variability

Staphylococci are involved in infections that follow diverse clinical scenarios ranging from acute to chronic, relapsing and therapy-refractive courses. Staphylococcal versatility, apart from their inherent virulence and antibiotic resistance, is grounded in phenotypic plasticity and ability to differentiate into specific subpopulations that enhance bacterial survival and persistence. Small colony variants (SCVs), persisters, and the biofilm cells undergo phenotypic, metabolic and genetic changes influencing the development of skill sets that are necessary to subvert or avoid external stressors during infection (DOI: 10.3390/antibiotics12020406).

Liu et al. conducted the first retrospective and a multi-case study on cardiac implantable electronic device (CIED) infections associated with staphylococcal SCVs. Thirty isolates were cultivated from 19 (21%) out of 90 culture-positive patients. *S. epidermidis* was the predominant (78.9%) SCV species. Comparative genomic analysis of the wild-type (WT) and the co-cultured *S. epidermidis* SCVs revealed that approximately one third of genes harboring mutations were associated with the cell wall. These genes are known to encode for proteins involved in bacterial adherence, biofilm formation, peptidoglycan hydrolysis, and cell division. The association between SCVs and clinical characteristics of CIED infections was also investigated by the authors. Patients with SCVs had a longer primary pacemaker implantation time, were more likely to have a history of the CIED replacement and infection, and had longer hospital stays. In addition, upregulated inflammatory responses, including a higher neutrophil percentage, were observed in patients with SCVs.

The SCV subpopulations demonstrate atypical characteristics including prolonged growth, biofilm formation, antibiotic tolerance, and enhanced intracellular persistence. These traits favor chronicization of infections and impede the efficacy of conventional therapeutic strategies based on the administration of antibiotics (Onyango and Liang).

Onyango and Liang analyzed the available literature regarding the effectiveness of Manuka honey (MH) as an alternative antimicrobial agent. Given the increase in antibiotic resistance among *Staphylococcus* species, including their SCV phenotypes, alternative treatment strategies are crucial. The research shows that Manuka honey, characterized by a high content of methylglyoxal, has significant antibacterial properties. The authors systematically reviewed the existing literature, emphasizing that Manuka honey effectively inhibits the development of various strains of *Staphylococcus*, including methicillin-resistant *Staphylococcus aureus* (MRSA). The impact of honey on SCVs was also discussed. According to the available literature data, Manuka honey has multifactorial antimicrobial properties, which include destroying bacterial cell walls and inhibiting biofilm formation, making it a promising alternative or complement to conventional antibiotics. This non-antibiotic approach could potentially alleviate the antibiotic resistance crisis by offering effective treatments against resistant bacterial strains without contributing to the development of further resistance.

In another study, Liang et al. tested MH, in a combination with gentamicin (G), rifampicin (R), and vancomycin (V) against three WT staphylococcal (*S. aureus*, *S. epidermidis*, and *S. lugdunensis*) strains and their SCV counterparts that were induced following exposure to the three antibiotics. The study demonstrated that a combination of MH with G, R, or V yields additive or partially synergistic activities against the WT strains. Importantly, the MH was shown to inhibit the *in vitro* development of antibiotic-induced SCVs. Moreover, MH added to cultures of stable SCVs contributed to the inhibition of SCVs growth, indicating that this substance is also able to prevent subsequent SCV proliferation. The obtained results hold a promise for the future application of MH to hamper the growth of SCVs, especially in patients exposed to a prolonged antibiotic therapy, which is known to induce the formation of these subpopulations.

Sharing ecological niches and iron limitation are among factors that can induce metabolic changes in bacteria, including production of iron-scavenging molecules (siderophores) and phenotypic differentiation. The study of Rajapitamahuni et al. reports that interactions between bacterial species influence the production of siderophores which is important to gain competitive advantages in microbial communities. Adaptive strategies of *S. aureus* under iron-rich and -defective conditions were investigated by the induction of interspecies interactions during co-cultivation with *Escherichia coli*, *Pseudomonas aeruginosa*, and *S. epidermidis*. Microbial phenotypes were examined at the molecular level using Raman spectroscopy. The glycerol alanine salts (GAST) medium was optimized for the analysis of siderophore production. The authors suggested a crucial role of microbial interactions and siderophore dynamics in directing phenotypic differentiation of *S. aureus*, especially under iron-deficient conditions. Bacterial co-culturing in the iron-rich environments had a high impact on *S. aureus* phenotype (evidenced by shifts in the Raman peaks or bands related to nucleic acids, proteins, amino acids, and lipids) irrespective of the co-cultured bacterial species. Iron depletion, in turn, revealed that *S. aureus* phenotypic differentiation is influenced by the interacting species. Adaptation to low-resource environments requires production of siderophores that outcompete other microorganisms in the pursuit of iron. When co-cultured under low-iron conditions with *E. coli*, *S. aureus* showed significant shifts in peaks associated with proteins/lipids, amino acids, nucleic acids, and proteins. The authors speculated that these shifts can correspond to membrane remodeling due to alterations in siderophore transport mechanisms, shifts in the metabolic flux toward different siderophore types, modifications in the gene expression profiles related to siderophore production/transport as well as changes in the enzymatic background required for the siderophore biosynthesis, respectively. Diverse siderophore production patterns (carboxylate, catecholate, and hydroxamate), depending on the interacting species, were observed in *S. aureus* under iron-rich conditions. Interestingly, siderophore production ceased completely following co-cultivation with *P. aeruginosa* although *S. aureus* showed the highest viability when co-cultured with this species. Possible explanations for halting the iron-scavenging molecules production in *S. aureus* included preservation of resources in response to competitive pressures or availability of siderophores produced by *P. aeruginosa*. It, therefore, can be concluded that bacterial adaptation to challenging conditions requires flexibility to use dual strategies associated with biosynthesis on the one hand, and piracy, on the other. In a broad sense, phenotypic differentiation can influence microbial pathogenicity and the host-pathogens interactions that will be important to determine the outcome of an infectious process.

## Author contributions

AB: Writing – original draft. AM: Writing – original draft.

